# CAP1 is overexpressed in human epithelial ovarian cancer and promotes cell proliferation

**DOI:** 10.3892/ijmm.2015.2089

**Published:** 2015-02-04

**Authors:** MINHUI HUA, SUJUAN YAN, YAN DENG, QINGHUA XI, RONG LIU, SHUYUN YANG, JIAN LIU, CHUNHUI TANG, YINGYING WANG, JIANXIN ZHONG

**Affiliations:** 1 Department of Obstetrics and Gynecology, Affiliated Hospital of Nantong University, Nantong, Jiangsu 226001, P.R. China; 2 Department of Obstetrics and Gynecology, The First People’s Hospital of Jingmen, Jingmen, Hubei 448000, P.R. China; 3 Departments of Pathology, Nantong University Cancer Hospital, Nantong, Jiangsu 226001, P.R. China; 4 Departments of Oncology, Nantong University Cancer Hospital, Nantong, Jiangsu 226001, P.R. China; 5 Department of Pathogen Biology, Medical College, Nantong University, Nantong, Jiangsu 226001, P.R. China

**Keywords:** adenylate cyclase-associated protein 1, human epithelial ovarian cancer, proliferation, therapeutic target

## Abstract

Adenylate cyclase-associated protein 1 (CAP1) regulates both actin filaments and the Ras/cAMP pathway in yeast, and has been found play a role in cell motility and in the development of certain types of cancer. In the present study, we investigated CAP1 gene expression in human epithelial ovarian cancer (EOC). Western blot analysis and immunohistochemistry were performed using EOC tissue samples and the results revealed that CAP1 expression increased with the increasing grade of EOC. In the normal ovarian tissue samples however, CAP1 expression was barely detected. Using Pearson’s χ^2^ test, it was demonstrated that CAP1 expression was associated with the histological grade and Ki-67 expression. Kaplan-Meier analysis revealed that a higher CAP1 expression in patients with EOC was associated with a poorer prognosis. In *in vitro* experiments using HO-8910 EOC cells, the expression of CAP1 was knocked down using siRNA. The proliferation of the HO-8910 cells was then determined by cell cycle analysis and cell proliferation assay using the cell counting kit-8 and flow cytometry. The results revealed that the loss of CAP1 expression inhibited cell cycle progression. These findings suggest that a high expression of CAP1 is involved in the pathogenesis of EOC, and that the downregulation of CAP1 in tumor cells may be a therapeutic target for the treatment of patients with EOC.

## Introduction

Epithelial ovarian cancer (EOC) is one of the most common types of ovarian tumors and is the leading cause of mortality among gynecological malignancies (1,2). Despite the rapid development of surgical and chemotherapy treatments, the 5-year survival rate for patients with EOC remains at approximately 30–50% due to the lack of effective early diagnostic methods. Thus, there is an urgent need for the identification of biological factors with sensitivity and specificity in order to improve prognosis and reduce the threat to the lives and health of women.

It is well known that cell physiology, including cell-cell junctions, cytoskeletal structure and cell morphology regulate cancer cell migration and invasion. The actin cytoskeleton plays a key role in cancer cell motility and metastasis, as its remodeling regulates a number of important cellular processes, such as cell adhesion, migration and morphological changes (3–5). Cyclase-associated proteins (CAPs) which exist in organisms from mammals to apicomplexan parasites (6) are a family of evolutionarily conserved proteins which are key to regulating actin dynamics and participate in Ras-mediated adenylyl cyclase activity (7,8). In mammal, cells have 2 CAP genes encoding the related CAP1 and CAP2 (9), both of which localize in the cell membrane and cytoplasm, and contain a C-CAP/co-factor C-like domain (10–13). The adenylate CAP1 gene, which encodes an actin monomer-binding protein is thought to facilitate processes, such as the establishment of cell polarity and mRNA localization (14). It also has the ability to control the cytoskeleton (15–19). The reorganization of the actin filament is regulated by actin-binding proteins in some signaling pathways that are essential for cell migration and several other intracellular events (20,21). CAP1 also plays an important role in actin filament turnover by effectively recycling cofilin and actin (22). On both ends of the actin filament, CAP1 rapidly translocates to the mitochondria upon treatment with agents that induce apoptosis (23). Studies have demonstrated that CAP1 is overexpressed in hepatocelluar carcinoma (24), breast cancer (25), lung cancer (26) and esophageal squamous cell carcinoma (27). However, to the best of our knowledge, no more information is available to date regarding the role of CAP1 in ovarian tumorigenesis.

Therefore, in the present study, we aimed to investigate the expression and function of CAP1 in EOC. We analyzed the expression of CAP1 protein in 119 EOC tissue specimens by immunohistochemistry and western blot analysis. We also determined the correlation of CAP1 expression with clinicopathological characteristics and evaluated the prognostic value of CAP1 in EOC by survival analysis. In addition, we knocked down CAP1 expression in order to explore the potential involvement of CAP1 in the regulation of cell proliferation. Our results indicate that CAP1 is a newly identified biomarker for EOC and may be used as a therapeutic target for the treatment of EOC in the future.

## Materials and methods

### Patients and tissue samples

All investigations described in this study were carried out after obtaining informed consent and in accordance with an Institutional Review Board protocol approved by the Partners Human Research Committee at the Affiliated Hospital of Nantong University, Nantong, China. A total of 119 human EOC tissue specimens (45 papillary serous adenocarcinoma, 16 papillary mucinous carcinoma, 14 endometrioid adenocarcinoma and 14 clear cell carcinoma specimens, as well as 30 specimens classified as ‘other’) were provided by the Department of Pathology, the Affiliated Hospital of Nantong University from 2004 to 2009. None of the patients had received chemotherapy or radiotherapy prior to surgery. A total of 10 tissue samples were obtained at the time of surgery and were immediately stored in liquid nitrogen and maintained at −80°C until used in western blot analysis, including 1 normal tissue sample from a woman who underwent hysterectomy for benign disease. The clinicopathological characteristics of all the participants are shown in Table I. According to the WHO system, the histological classification of tumors was graded as follows: well differentiated [grade 1 (G1); n=15], moderately differentiated [grade 2 (G2); n=33] and poorly differentiated [grade 3 (G3); n=71].

### Antibodies

The antibodies used for western blot analysis and immunohistochemistry were as follows: mouse anti-CAP1 monoclonal antibody (SC-376286), mouse anti-human Ki-67 monoclonal antibody (SC-101861), mouse anti-human proliferating cell nuclear antigen (PCNA) monoclonal antibody (sc-56), rabbit anti-human cyclin A polyclonal antibody (sc-751) and rabbit anti-human glyceraldehyde 3-phosphate dehydrogenase (GAPDH) polyclonal antibody (sc-25778; all from Santa Cruz Biotechnology, Inc., Santa Cruz, CA, USA).

### Western blot analysis

Total protein extracts (100 mg) were subjected to 10% sodium dodecyl sulfate-polyacrylamide gels electrophoresis (SDS-PAGE) and then transferred onto polyvinylidene difluoride (PVDF) filter membranes (Millipore, Bedford, MA, USA). After the membranes were blocked in 5% non-fat milk in TBST (150 mM NaCl, 20 mM Tris, 0.05% Tween-20) for 2 h, they were incubated with the primary antibodies overnight at 4°C. After washing the membranes with TBST 3 times for 5 min each, horseradish peroxidase-linked IgG secondary antibodies (donkey anti-mouse IgG-HRP, sc-2314; Santa Cruz Biotechnology, Inc.) were added followed by incubation for 2 h at room temperature. The membranes were developed using the ECL detection system.

### Immunohistochemistry

Serial sections (5-*μ*m-thick) were mounted on glass slides coated with 10% polylysine and were dewaxed in xylene and rehydrated in graded ethanol. Endogenous peroxidase activity was blocked by soaking in 0.3% hydrogen peroxide. The sections were then processed in 10 mmol/l citrate buffer (pH 6.0) and heated to 121°C in an autoclave for 20 min to retrieve the antigen. After rinsing in phosphate-buffered saline (PBS, pH 7.2), the sections were incubated with mouse anti-human CAP1 antibody (diluted 1:10,000) and mouse anti-human Ki-67 antibody (diluted 1:600) for 2 h at room temperature. Negative control slides were also processed in parallel using a non-specific immunoglobulin IgG (Santa Cruz Biotechnology, Inc.) at the same concentration as the primary antibody. All slides were processed using the peroxidase-anti-peroxidase method (Dako, Hamburg, Germany). Following rinsing with PBS, the peroxidase reaction was visualized by incubating the sections with the liquid mixture, DAB (0.1% phosphate buffer solution, 0.02% diaminobenzidine tetrahydrochloride and 3% H_2_O_2_). After rinsing in water, the sections were counterstained with hematoxylin, dehydrated and cover-slipped. All the immunostained sections were observed under a Leica fluorescence microscope (Leica Microsystems, Wetzlar, Germany).

For the assessment of CAP1 and Ki-67, at least 10 high-power fields in each specimen were randomly selected, and the percentage of cells with nuclear and cytoplasmic staining was examined with a total number of >500 cells counted to determine the labeling index in a single section. Tissues with no staining were rated as 0, with a faint staining or moderate to strong staining in ≤25% of cells as 1, with moderate staining or strong staining in 25–50% of cells as 2, and strong staining in ≥50% of cells as 3. For statistical analysis, a score of <2 was counted as low expression, while a score of ≥2 was counted as overexpression, as previously described (24). When evaluating Ki-67 protein immunoreaction, staining was scored in a semi-quantitative manner. A cut-off value of 50.7% or more positively stained nuclei in 5 high-power fields were used to identify Ki-67 staining as follows: the high expression group (≥50.7%) and low expression group (<50.7%). All the immunostained sections were evaluated in a blinded manner without knowledge of the clinicopathological variables of the patients. In half of the samples, staining was repeated twice to avoid technical errors, but similar results were obtained in these samples.

### Cell culture

The human EOC cells, HO-8910, were purchased from the Shanghai Institute of Cell Biology and cultured in RPMI-1640 supplemented with 10% heat-inactivated fetal calf serum and 100 U/ml penicillin-streptomycin mixture (both from Gibco-BRL, Grand Island, NY, USA) at 37°C and 5% CO_2_.

### Cell cycle analysis

Cells were harvested at the proper time and fixed in 70% ethanol overnight at 4°C and then incubated with 1 mg/ml RNase A for 30 min at 37°C. Subsequently, the cells were stained with propidium iodide (PI, 50 mg/ml; Becton-Dickinson, San Jose, CA, USA) in PBS, 0.5% Tween-20, and analyzed using a flow cytometer BD FACScan (Becton-Dickinson, San Jose, CA, USA) as well as CellQuest acquisition and analysis programs. As regards cell synchronization, serum deprivation was used for cell cycle G1-S phase arrest.

### siRNA and transfection

Duplex siRNAs were synthesized as follows by Biomics Biotechnologies Co., Ltd. (Nantong, China): The sequences were as follows CAP1-specific, 5′-GAAAUGAA UGAUGCCAdTdT-3′ and control, 5′-UGGCGGCAUUCAUU UCdTdT-3′. The HO-8910 cells were seeded the day prior to transfection at a confluence of 50–70%. In addition, a Mock group with no transfection was also used as a control. The transient transfection of CAP1 and non-specific siRNA oligos was carried out using Lipofectamine 2000 (Invitrogen, St. Louis, MO, USA) in accordance with the manufacturer’s instructions. The cells were harvested 48 h after transfection and used for the experiment. The experiments were repeated at least 3 times.

### Cell proliferation assay

The cell counting kit-8 (CCK-8) was used to measure cell proliferation according to the manufacturer’s instructions (Dojindo, Kumamoto, Japan). The cells were seeded into a 96-well cell culture cluster plates (Corning, Inc., Corning, NY, USA) at a concentration of 2×10^4^ cells/well in a volume of 100 *μ*l culture medium and grown overnight. CCK-8 reagents (Dojindo) were then added to each well followed by incubation for an additional 2 h at 37°C. The absorbency was measured at a test wavelength of 490 nm and a reference wavelength of 630 nm using a microplate reader (Bio-Rad, Hercules, CA, USA). The experiments were repeated at least 3 times.

### Statistical analysis

All statistical analyses were performed using SPSS 17.0 statistical software. The association between CAP1 and Ki-67 expression and clinicopathological characteristics was analyzed using Pearson’s χ^2^ test. Multivariate analysis was performed using Cox’s proportional hazards model. CAP1 and Ki-67 expression was quantified using Pearson’s correlation co-efficient. Kaplan-Meier analysis was used to evaluate the survival curve, and the log-rank test was performed for analysis.

## Results

### CAP1 is overexpressed in EOC tissues

**Figure 1:**
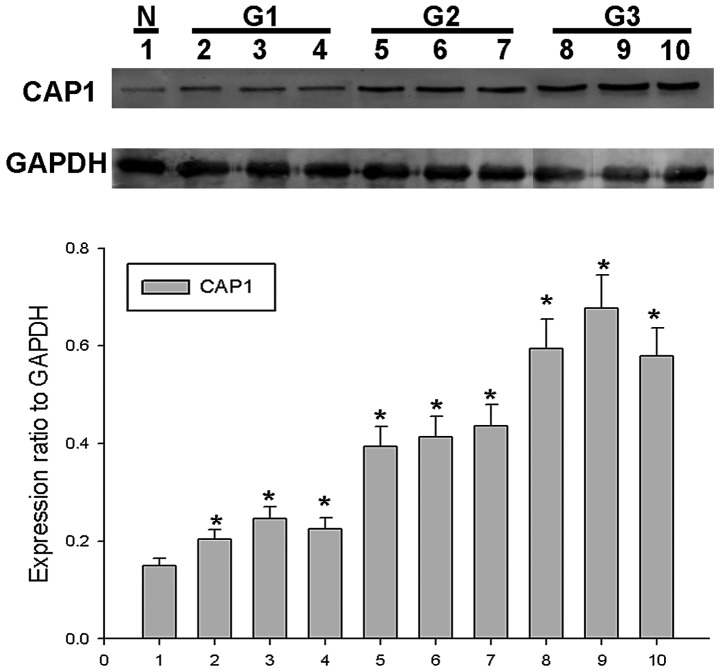
Cyclase-associated protein 1 (CAP1) is expressed in epithelial ovarian cancer (EOC) tissues. Expression of CAP1 in 1 normal tissue specimen (N) and 9 cancer tissue specimens from 3 tumors classified as grade 1 (G1) to grade 3 (G3) (3 tissues from each tumor). GAPDH was used as a control for protein loading and integrity. The same experiment was repeated at least 3 times. ^*^P<0.05, compared with normal tissue.

**Figure 2:**
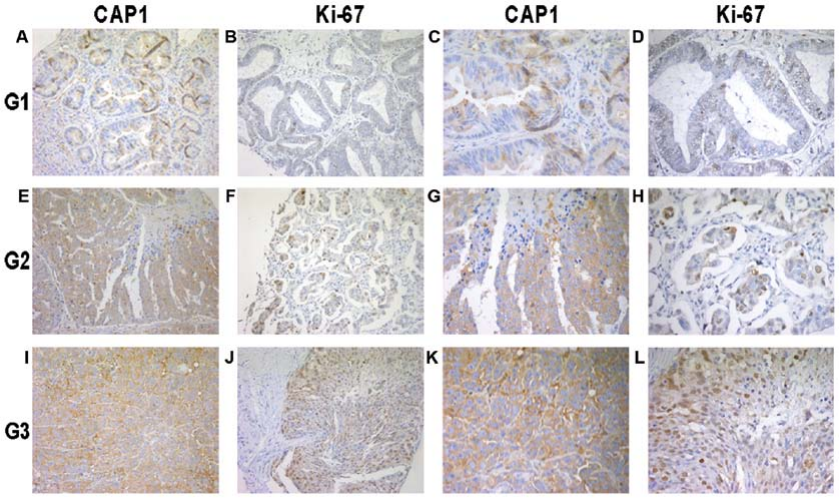
Immunohistochemical analysis of cyclase-associated protein 1 (CAP1) and Ki-67 expression in the 119 epithelial ovarian cancer (EOC) tissue specimens. Paraffin-embedded tissue sections were stained with antibodies to CAP1 and Ki-67 and counterstained with hematoxylin. (A-D) CAP1 and Ki-67 immunoreactivity in cancer tissue from a tumor classified as grade 1 (G1). (E-H) CAP1 and Ki-67 staining in cancer tissue from a tumor classified as grade 2 (G2). (I-L) CAP1 and Ki-67 staining in cancer tissue from a tumor classified as grade 3 (G3). The experiment details were described in ‘Materials and methods’. (A, B, E, F, I and J) images at magnification, x200. (C, D, G, H, K and L) images at magnification, x400.

To confirm the role of CAP1 in EOC, western blot analysis for CAP1 was performed using surgical specimens. We found that CAP1 protein expression gradually increased in 9 EOC tissues from 3 tumors classified as G1 to G3 (3 tissues from each tumor) in comparison with 1 normal ovarian tissue sample in which CAP1 expression was barely detected (Fig. 1). In order to determine the role of CAP1 in the progression and development of EOC, we examined the intracellular expression of CAP1 and Ki-67 in 119 specimens of EOC by immunohistochemical analysis. We found that CAP1 was mainly located in the cytoplasm of the EOC cells (Fig. 2). The high expression of CAP1 was accompanied by the high expression of Ki-67 localized in the nucleus. In addition, CAP1 was highly expressed in the poorly differentiated tumor specimens compared to the well differentiated ones; similar results were obtained for Ki-67 expression.

### Correlation of CAP1 expression with clinicopathologic characteristics in EOC

**Figure 3:**
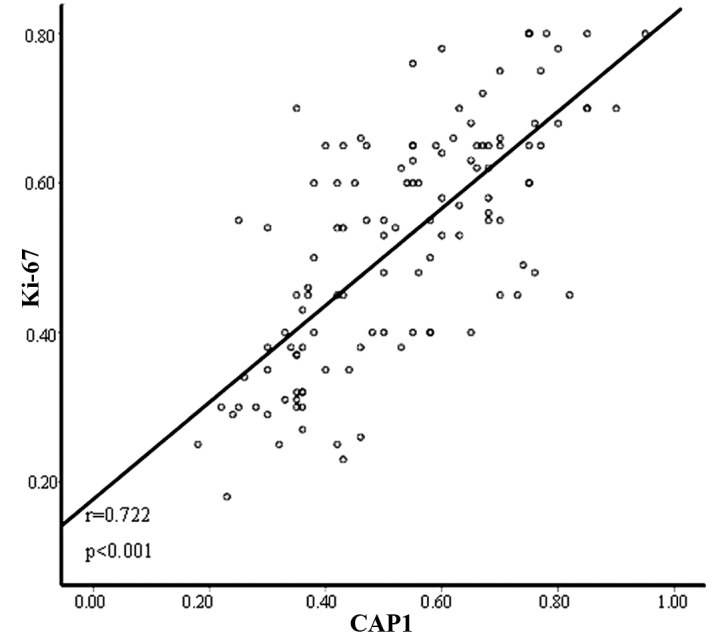
Correlation between cyclase-associated protein 1 (CAP1) and Ki-67 expression in epithelial ovarian cancer (EOC). Scatter plot of CAP1 against Ki-67 with the regression line showing a correlation between them using Spearman’s correlation co-efficient.

To clarify the clinicopathological significance of CAP1, we analyzed the correlation of CAP1 expression with the clinicopathologic characteristics of patients with EOC (Table I). We divided the tumor specimens into 2 groups according to the expression of CAP1. CAP1 expression was significantly associated with the histological grade (P=0.010) and Ki-67 expression (P<0.001), whereas there was no correlation observed with the FIGO stage (P=0.477), menopause (P=0.395), ascites (P=0.666), lymph node status (P=0.209), malignant tumor cells (P=0.472), metastases to other organs (P=0.666), age (P=0.775) and histologic subtype (P=0.071). In addition, Pearson’s correlation co-efficient revealed that there was a positive correlation between CAP1 expression and Ki-67 expression (r=0.722, P<0.001) (Fig. 3).

### Expression of CAP1 and prognosis of EOC

**Figure 4:**
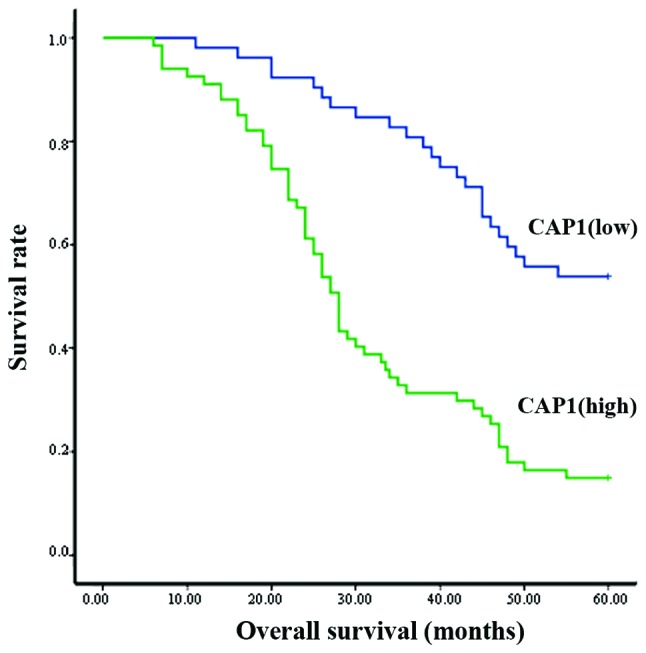
Kaplan-Meier survival curves demonstrate the correlation between survival and the expression of cyclase-associated protein 1 (CAP1) in 119 patients with epithelial ovarian cancer (EOC). Patients with a lower CAP1 expression had a longer survival than those with a higher CAP1 expression.

Kaplan-Meier analysis revealed that out of the 119 clinical cases examined, the patients with a high expression of CAP1 had a poorer overall survival than those with a lower expression (P<0.001; Fig. 4). Pearson’s χ^2^ test indicated that the histological grade (P=0.047), CAP1 (P<0.001) and Ki-67 (P<0.001) were independent prognostic indicators of EOC (Table II). Moreover, multivariate analysis using Cox’s proportional hazards model showed that the histological grade (P<0.001), CAP1 (P= 0.029) and Ki-67 (P<0.001) were independent prognostic indicators for patient overall survival (Table III). The survival curve indicated that the median survival for patients with a high CAP1 expression was significantly shorter than the median survival for those patients with a low CAP1 expression (Fig. 4).

### CAP1 expression and cell cycle progression

**Figure 5:**
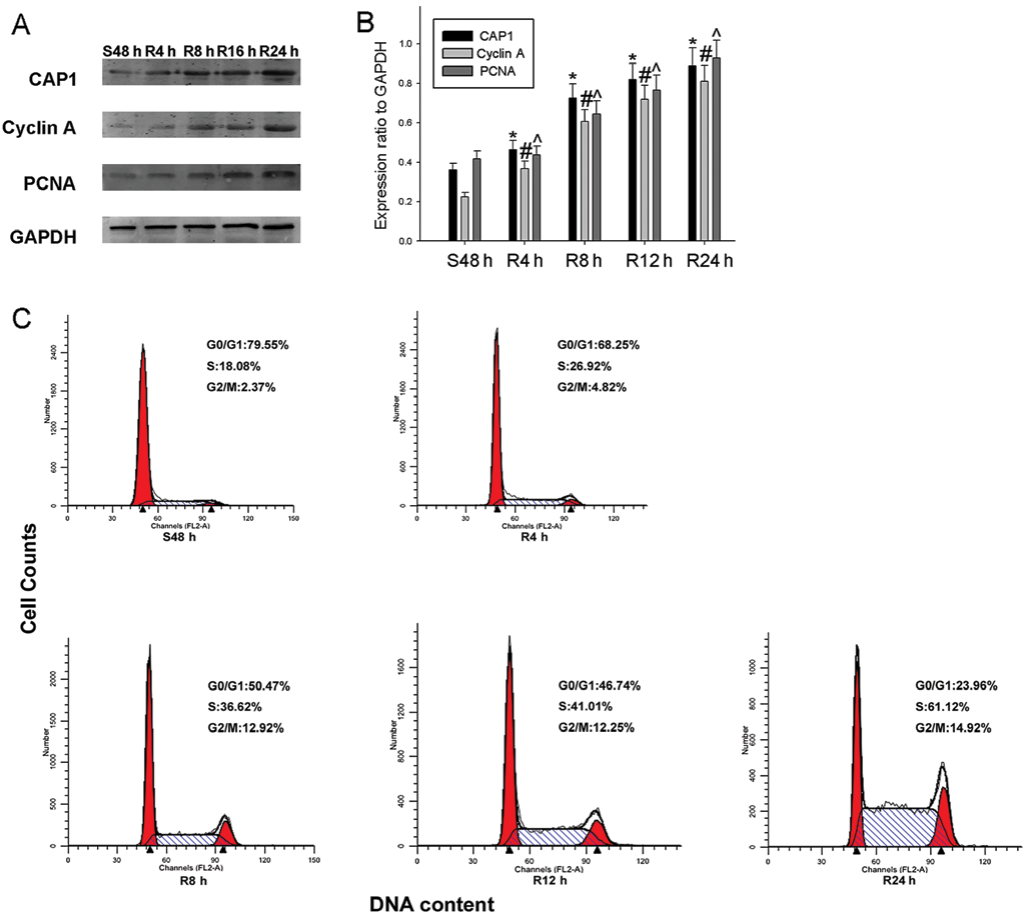
Expression of cyclase-associated protein 1 (CAP1) and cell cycle-related molecules in proliferating HO-8910 cells. (A) Cells were serum-starved for 48 h and following serum re-feeding, cell lysates were prepared and analyzed by western blot analysis using antibodies directed against CAP1, proliferating cell nuclear antigen (PCNA) and cyclin A. GAPDH was used as a control for protein loading and integrity. (B) The histogram demonstrates the ratio of CAP1, PCNA and cyclin A protein to GAPDH for each time point by densitometry. (C) Flow cytometric quantification of cell cycle progression in HO-8910. The cells were serum-starved for 48 h, and medium containing 10% fetal bovine serum (FBS) was then added for the indicated periods of time (0, 4, 8, 12 and 24 h). Data are the means ± standard error of the mean (SEM) of 3 independent experiments. n=3, ^*,#,^^P<0.05, compared with control cells serum-starved for 48 h.

It has been reported that the knockdown of the expression of CAP1 affects the breast cancer cell cycle, inhibiting the growth of breast cancer cancer (25). In this study, we found that a high CAP1 expression associated with a poor prognosis of patients with EOC. Thus, we hypothesized that CAP1 participates in the cell cycle of HO-8910 cells. We subjected the cells to serum starvation and serum re-feeding and found that the expression of CAP1 gradually increased, as well as that of the proliferation markers, PCNA and cyclin A, during cell progression. Flow cytometric analysis revealed that the HO-8910 cells subjected to serum deprivation for 48 h were arrested in the G1 phase, and following serum re-feeding, the population of HO-8910 cells in the S phase increased from 18.08 to 61.12% (Fig. 5C). To further validate these results, we collected HO-8910 cellular protein at different time points, and western blot analysis was performed to determine whether CAP1 expression is cell cycle-dependent in HO-8910 cells. We found that CAP1 expression was significantly increased as early as 4 h following serum re-feeding in the HO-8910 cells. The expression of the cell proliferation markers, PCNA and cyclin A, showed a similar tendency (Fig. 5A and B).

### Knockdown of CAP1 inhibits the proliferation of HO-8910 cells

**Figure 6:**
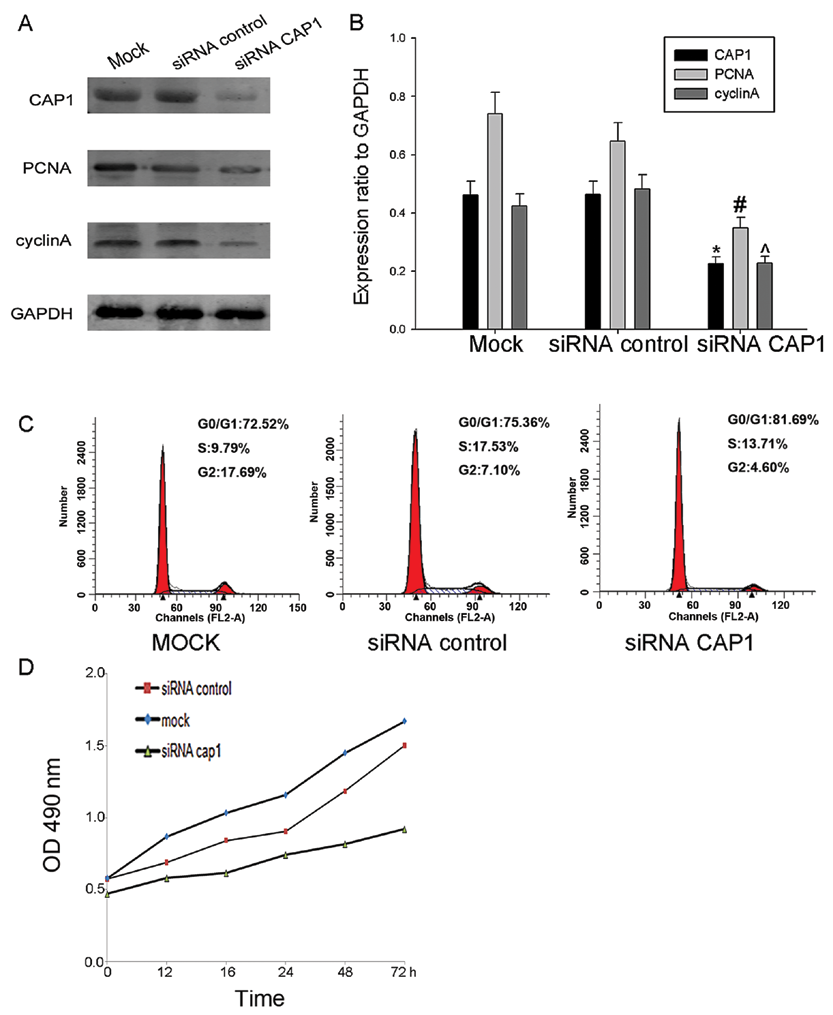
Knockdown of the expression of cyclase-associated protein 1 (CAP1) inhibits cell proliferation. (A) Western blot analysis was used to determine the levels of cyclin A and proliferating cell nuclear antigen (PCNA) in the HO-8910 cells following the depletion of CAP1. Transfection with siRNA led to a decrease in CAP1 expression, as well as in PCNA and cyclin A expression, as shown by western blot analysis 48 h following the transfection of HO-8910 cells. (B) Histogram demonstrates the ratio of CAP1, PCNA and cyclin A protein to GAPDH at each time point by densitometry. (C) Cell cycle analysis following the downregulation of CAP1 by siRNA targeting CAP1 in HO-8910 cells. Following transfection of the HO-8910 cells with siRNA targeting CAP1, an increase in the cell number at the G0/G1 phase was observed, with a concomitant decrease in the number of cells in the S phase, as shown by flow cytometry. (D) Comparison of growth curves between the cells transfected with siRNA targeting CAP1 and control siRNA and mock-transfected cells using the cell counting kit-8 (CCK-8). The data are the means ± standard error of the mean (SEM) (n=3; ^*,#,^^P<0.05, compared with the control- and mock-transfected cells). Each experiment was repeated at least 3 times.

To further determine the effect of CAP1 on cell proliferation, we knocked down endogenous CAP1 in HO-8910 cells using siRNA targeting CAP1 and the effects to the cells transfected with control siRNA and mock siRNA (Fig. 6A and B). The knockdown of CAP1 using siRNA inhibited the accumulation of cyclin A and PCNA compared with control- and mock-transfected cells (Fig. 6A and B). Additionally, we found that the proliferation rate of the HO-8910 cells transfected with siRNA exhibited a decrease compared with the cells transfected with the control siRNA and the mock-transfected cells (Fig. 6D). From these results, we ascertained that CAP1 plays a positive role in the regulation of cell proliferation.

## Discussion

CAP1, a member of the CAP family in mammalian cells, was first identified as a component of the yeast adenylyl cyclase complex and conserved in all eukaryotic organisms. The CAP family contains 4 highly conserved protein domains, one of which is the N-terminal which interacts with adenylyl cyclase and induces the activity of RAS following exogenous signals, whereas the C-terminal half of CAPs is involved in the cell differentiation and depolymerization of the F-actin filamentactin (28). As a monomeric actin binding protein, CAP is involved in cell polarization, the distribution of actin filaments and mRNA in a *Dictyostelium* (29). The expression of CAP is associated with an abnormally large cell size, random budding pattern and an abnormal actin distribution in yeast (30,31).

A number of scholars have started to investigate the association between CAP1 and cancer. CAP1 has been to be commonly overexpressed in pancreatic cancers, and its level in clinical cases has been shown to be associated with neuronal invasion and lymph node metastasis. The knockdown CAP1 has been shown to reduce cell motility and migration (32). It has been reported that CAP1 is upregulated in breast cancer. After knocking down its expression, the proliferation and migration of MDA-MB-231 cells was shown to decrease, inducing changes in morphology, which were associated with the arrangement of F-actin (25). Western blot analysis, real-time PCR and immunohistochemical analysis have been used to prove that CAP1 is overexpressed in hepatocellular carcinoma compared with adjacent non-cancerous liver tissues, and that it is positively associated with HCC cell metastasis (24). CAP1 overexpression has been shown to be significantly associated with lymph node status in esophageal squamous cell carcinoma. The knockdown of CAP1 in TE1 cells has been shown to result in a decreased migration capability and the overexpression of CAP1 promotes TE1 cell migration (27). In this study, we found that CAP1 was overexpressed in EOC. Using immunohistochemistry, we found that CAP1 was highly expressed in poorly differentiated specimens compared to well differentiated ones, and similar results were obtained for Ki-67 expression. Kaplan-Meier analysis revealed that out of the 119 clinical cases, the patients with a high expression of CAP1 had a poorer overall survival than those with a lower CAP1 expression. In addition, the knockdown of CAP1 expression in an *in vitro* experiment revealed that the loss of CAP1 inhibit the proliferation of HO-8910 cells.

It has been demonstrated that the morphologic changes of malignant tumor cells enhance the migration capacity of the cells and lead to invasion and metastasis (33). To a certain degree, the maintainance and changes in the structure and function of cells are achieved by regulating the structure and function of the actin cytoskeleton, which is the key to the reorganization of the actin cytoskeleton. When stimulated, CAP regulates the polymerization and disassembly of downstream actin protein, thus affecting the growth and differentiation of cells. CAP1 also takes part in accelerating the turnover of actin filaments by the recycling of cofilin and actin on both ends of the actin filament (34). Therefore, we hyopthesized that the molecular mechanisms of action of CAP1 in the pathogenesis of EOC may involve its downstream actin protein. However, further studies are required to identify the precise signaling pathways involved.

As CAP1 is overexpressed in EOC, it may thus serve as a prognostic marker for EOC. Using western blot analysis, we found that the expression of CAP1 was higher in the 9 EOC tissues compared to the 1 normal tissue, in which CAP1 expression was barely detected. Furthermore, immunohistochemistry of the 119 paraffin-embedded tissue sections of EOC also revealed that CAP1 immunostaining was located in the cytoplasm and was upregulated in the poorly differentiated tumor cells compared to the well differentiated ones. These results were similar to those obtained for Ki-67 expression, highlighting that CAP1 expression significantly correlated with the proliferation of EOC cells. The association between CAP1 and markers of the cell cycle was then detected by a starvation-release experiment.

In conclusion, this study demonstrates that CAP1 is an independent prognostic factor in EOC. Our data suggest that CAP1 plays an important role in cell proliferation in EOC and that CAP1 may be a potential therapeutic target in EOC chemotherapy.

## Figures and Tables

**Table I:** Expression of CAP1 in the 119 human ovarian cancer specimens.

Clinicopathological characteristics	No. of cases	CAP1 expression		P-value^a^
		Low	High	
Age (years)				0.775
≤50	35	16	19	
>50	84	36	48	
FIGO stage, n (%)				0.477
I	38	18	20	
II	31	10	21	
III	33	15	18	
IV	17	9	8	
Histological grade, n (%)				0.010^b^
1	15	12	3	
2	33	13	20	
3	71	27	44	
Histological subtype, n (%)				0.071
Serous	45	20	25	
Mucinous	16	10	6	
Endometrioid	14	7	7	
Clear cell	14	8	6	
Others	30	7	23	
Menopause				0.395
Absent	43	21	22	
Present	76	31	45	
Lymph node status, n (%)				0.209
Negative	87	35	52	
Positive	32	17	15	
Ascites, n (%)				0.666
Absent	66	30	36	
Present	53	22	31	
Malignant tumor cells in peritoneal fluid, n (%)				0.472
Absent	90	41	49	
Present	29	11	18	
Metastases to other organs, n (%)				0.666
Absent	66	30	36	
Present	53	22	31	
Ki-67				<0.001^b^
Low expression	50	37	13	
High expression	69	15	54	

a Statistical analyses were performed using the Pearson’s χ^2^ test.

b P<0.05 indicates statistical significance. CAP1, cyclase-associated protein 1.

**Table II:** Survival status and clinicopathological characteristics in the 119 human ovarian cancer specimens.

Clinicopathological characteristics	Total	Survival status		P-value^a^
		Alive	Deceased	
Age (years)				0.099
≤50	35	15	20	
>50	84	23	61	
FIGO stage, n (%)				0.655
I	38	15	23	
II	31	8	23	
III	33	10	23	
IV	17	5	12	
Histological grade, n (%)				0.047^b^
1	15	5	10	
2	33	5	28	
3	71	28	43	
Histological subtype, n (%)				0.229
Serous	45	19	26	
Mucinous	16	3	13	
Endometrioid	14	5	9	
Clear cell	14	5	9	
Others	30	6	24	
Menopause				0.081
Absent	43	18	25	
Present	76	20	56	
Lymph node status, n (%)				0.217
Negative	87	25	62	
Positive	32	13	19	
Ascites, n (%)				0.051
Absent	66	26	40	
Present	53	12	41	
Malignant tumor cells in peritoneal fluid, n (%)				0.135
Absent	90	32	58	
Present	29	6	23	
Metastases to other organs, n (%)				0.051
Absent	66	26	40	
Present	53	12	41	
CAP1				<0.001^b^
Low expression	52	28	24	
High expression	67	10	57	
Ki-67				<0.001^b^
Low expression	50	34	16	
High expression	69	4	65	

a Statistical analyses were performed using the Pearson’s χ^2^ test.

b P<0.05 indicates statistical significance. CAP1, cyclase-associated protein 1.

**Table III:** Contribution of various potential prognostic factors to survival by Cox regression analysis of the 119 human ovarian cancer specimens.

	Hazard ratio	P-value	95.0% CI
Histological grade	0.552	<0.001^a^	0.396–0.770
CAP1 expression	0.548	0.029^a^	0.319–0.941
Ki-67 expression	6.161	<0.001^a^	3.256–11.655

a P<0.05 indicates statistical significance. CI, confidence interval; CAP1, cyclase-associated protein 1.
